# Correspondence

**Published:** 1869-11

**Authors:** Theodore Griffin

**Affiliations:** Troy, Mo.


### Correspondence.

PROF. DAVIS:

DEAR SIR:—The following case which recently came under my observation for treatment presents some interesting features, illustrative of the effect of long standing indigestion upon the nervous system:

Mr. P., aged 50 years, of great muscular development and strength, by occupation a tradesman, has for 15 years suffered with the ordinary symptoms of dyspepsia, and some three months ago, he had an attack of remittant fever: aside from this, his health has for years been good, until some two months ago, when he was suddenly seized with a sort of sinking or fainting spell, which continued some five minutes; consciousness entirely suspended, with a feeling of oppression and mental debility, with partial loss of memory.

Attacks like this have since continued to occur, sometimes five or six a day, and again disappearing for three or four days, only to return again with more unpleasant results, reducing his physical strength, and greatly impairing the strength and activity of his mind. It was upon one of these returns of attacks that I was consulted, and asked for an opinion as to the pathological condition of the gentleman. Upon examination, I found him to be a man of some 170 or 180 ℔s. weight, compactly built, with a moderate amount of adipose tissue; complexion rather florid; temperature of the skin above normal; mental depression, with much anxiety concerning his condition, and apprehensive that he was becoming demented. Can not continue a connected train of thought; suffers no pain; sleeps ordinarily well; circulation good; pulse full; tongue coated with a heavy, white coat in the centre; appetite good generally, sometimes voracious; bowels regular. The "spells" of which he complains occur in the daytime, but at no particular period of the day.

I believe that the symptoms detailed result from functional derangement of the nervous system, resulting from an impoverished state of the blood, depending primarily upon imperfect digestion, and deficient nutrition and assimilation.

The attacks do not resemble epilepsy in any particular. The patient for a long time believed that his stomach was infested with a reptile of some kind, and said that the "spells" were preceded and induced by the reptile making an effort to get into his throat, and that when it reached a certain point in his throat the attack came on. He was accordingly treated for tape-worm, and has undergone a variety of treatment, general and alterative—but all has availed nothing.

He is now upon the following:—

℞. Brom. Potass, ---------------------------- ʒij.

Mur. Tr. Fe., ---------------------------- ʒj.

Quinia, ---------------------------------- gr.xvi.

Syr. simplex ℥j., misce; of this he takes one teaspoonful half-hour after meals, with a pill of rhei. every morning. I think he is being benefited by this treatment.

I desire an expression of opinion from the profession, as to the pathological condition of this gentleman; if I have detailed his condition with sufficient conciseness to render such an expression possible.

THEODORE GRIFFIN, M.D.

*Troy, Mo.*

---

